# Anesthetic Management of Recurrent Renal Cell Carcinoma With Complete Obstruction of Inferior Vena Cava (IVC): A Case Report

**DOI:** 10.7759/cureus.87079

**Published:** 2025-06-30

**Authors:** Sherin Abdelhamid, Khaled S Abuamra, Ahmad Nabil, Hatem Ibrahim, Fariborz Bagheri, Cornelia Weidinger

**Affiliations:** 1 Department of Anesthesia, Dubai Hospital, Dubai Health, Dubai, ARE; 2 Department of Urology, Dubai Hospital, Dubai Health, Dubai, ARE

**Keywords:** anesthetic management, case report, inferior vena cava obstruction, renal cell carcinoma, vascular surgery

## Abstract

We report the perioperative management of a 67-year-old woman with recurrent renal cell carcinoma (RCC) complicated by complete inferior vena cava (IVC) obstruction, confirmed intraoperatively, who underwent en bloc resection of the tumor and involved segment of the IVC. Significant anesthetic challenges included stage 4 chronic kidney disease (CKD), intraoperative hemodynamic instability related to tumor manipulation, and the need to anticipate potential physiological effects of IVC clamping and unclamping. A multimodal anesthetic strategy including general anesthesia, bilateral rectus sheath block, invasive monitoring, and individualized fluid and vasopressor therapy allowed for responsive management of hemodynamic fluctuations. Postoperatively, the patient had an uncomplicated recovery in the intensive care unit. This report highlights the importance of meticulous perioperative planning, intraoperative adaptability, and multidisciplinary coordination in managing complex and evolving oncovascular scenarios.

## Introduction

Renal cell carcinoma (RCC) accounts for approximately 2-3% of adult malignancies and has a well-documented propensity to extend into the venous system, including the inferior vena cava (IVC), in 4-10% of cases [1]. Surgical management of RCC with IVC thrombus poses significant technical and anesthetic challenges, especially when complete IVC obstruction is present. These cases require complex vascular control and multidisciplinary coordination due to the risks of massive bleeding, hemodynamic instability, and venous air embolism [2].

From an anesthetic standpoint, complete IVC occlusion introduces unique considerations, including dynamic changes in preload, the potential for embolic events during tumor handling, and the need for continuous hemodynamic monitoring [3]. These challenges are further amplified in patients with comorbidities, such as chronic kidney disease (CKD), hypertension, and diabetes mellitus. Anesthetic strategies must therefore incorporate vigilant cardiovascular monitoring, judicious fluid management, and opioid-sparing analgesia to mitigate perioperative complications.

Recent evidence supports the use of balanced anesthesia combined with regional techniques to enhance perioperative outcomes in high-risk surgical populations. Multimodal analgesia incorporating rectus sheath block has been shown to reduce intraoperative opioid consumption, facilitate hemodynamic stability, and improve postoperative recovery [4]. Furthermore, adherence to enhanced recovery after surgery (ERAS) protocols, including early extubation and reduced ICU length of stay, has demonstrated favorable outcomes in major abdominal and vascular surgery [5].

This report describes the anesthetic management of a patient with recurrent RCC and complete infrahepatic IVC obstruction undergoing en bloc tumor and IVC resection. We highlight the integration of regional anesthesia, balanced anesthetic techniques, and hemodynamic monitoring in achieving early extubation, minimal ICU stay, and complication-free recovery in a high-risk oncovascular setting.

## Case presentation

**Figure 1:**
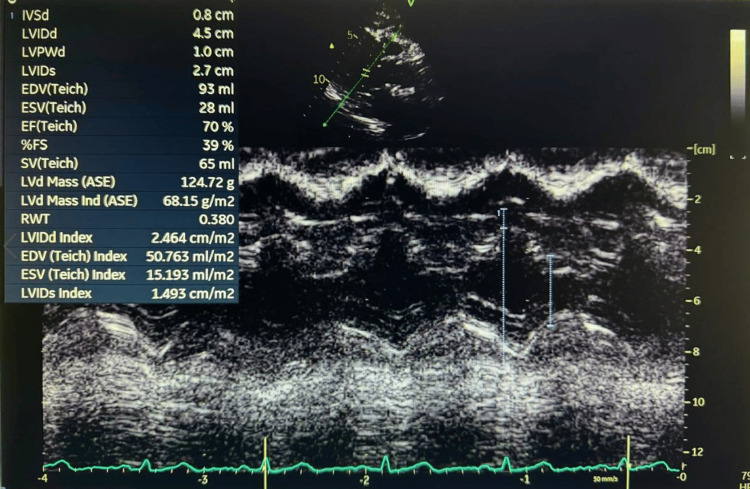
M-mode transthoracic echocardiography demonstrating preserved left ventricular systolic function. The calculated ejection fraction (EF) is 70%, and the fractional shortening (FS) is 39%, indicating normal contractility. Left ventricular dimensions and wall thicknesses are within normal reference ranges. IVSd: interventricular septal thickness in diastole; LVIDd: left ventricular internal diameter in diastole; LVPWd: left ventricular posterior wall thickness in diastole; LVIDs: left ventricular internal diameter in systole; EDV: end-diastolic volume; ESV: end-systolic volume; EF: ejection fraction; FS: fractional shortening; SV: stroke volume; LVd: left ventricular diameter; ASE: American Society of Echocardiography; RWT: relative wall thickness

**Figure 2:**
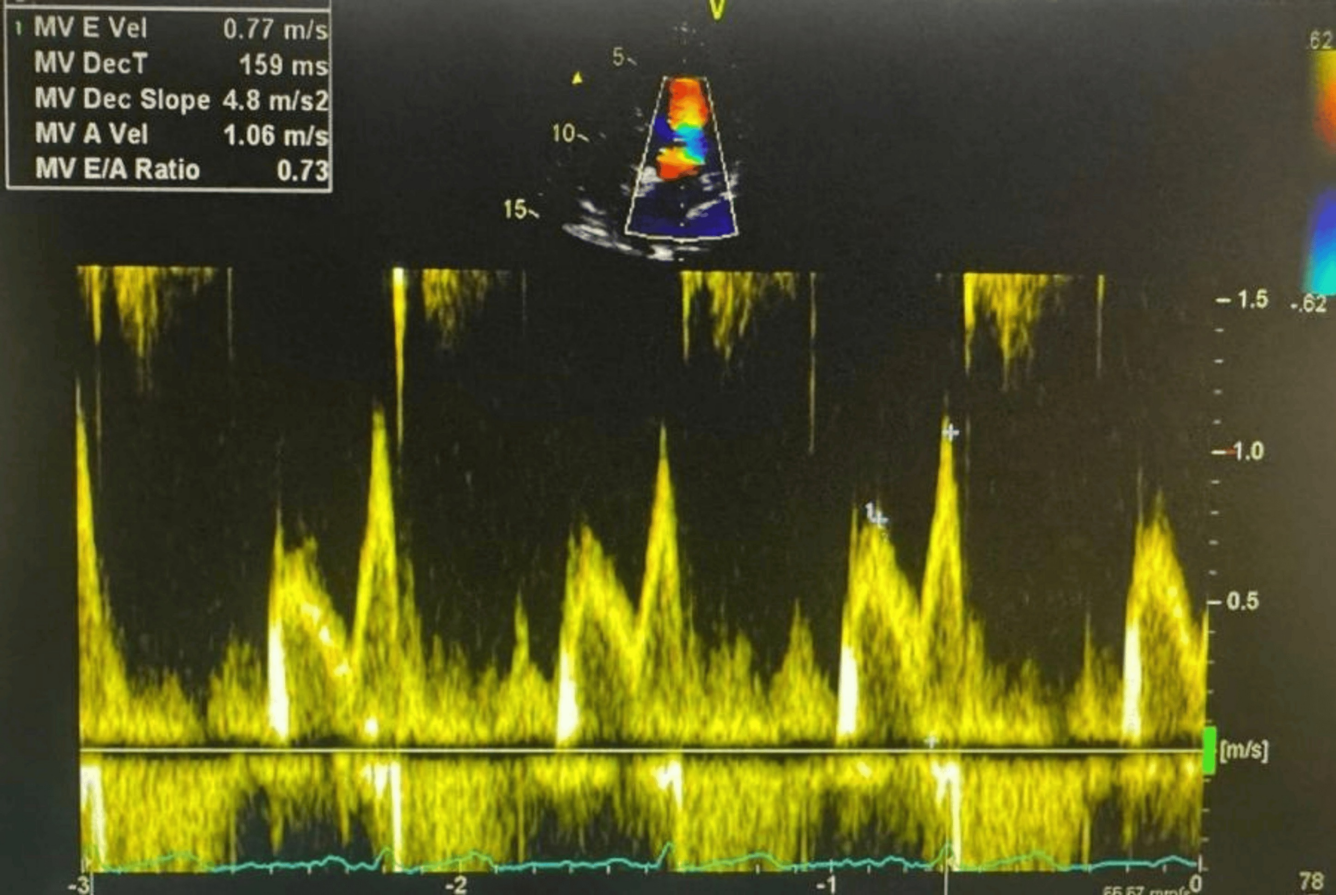
Pulse-wave Doppler echocardiography of mitral inflow showing an E/A ratio of 0.73 (E=0.77 m/s, A=1.06 m/s), consistent with grade I diastolic dysfunction (impaired relaxation pattern). MV E Vel: mitral valve E-wave velocity; MV DecT: mitral valve deceleration time; MV A Vel: mitral valve A-wave velocity; MV E/A ratio: mitral valve E/A ratio

A 67-year-old woman (weight: 83.7 kg, height: 153 cm, BMI: 35.76 kg/m²) with a history of right nephrectomy for clear renal cell carcinoma (RCC) (pT3aN1, grade IV), insulin-dependent type 2 diabetes mellitus, hypertension, and stage 4 chronic kidney disease (eGFR: 17.1 mL/min/1.73 m²) was scheduled for resection of a recurrent renal mass. She was undergoing immunotherapy prior to surgery. The patient reported no current cardiac or respiratory symptoms and maintained an acceptable level of functional capacity. Despite complete infrahepatic IVC obstruction, the patient remained clinically asymptomatic, likely due to the gradual progression of the lesion. Her baseline vital signs were stable: blood pressure was 144/61 mmHg, heart rate 73 beats per minute, and oxygen saturation 98% on room air. Preoperative transthoracic echocardiography showed preserved left ventricular systolic function with an estimated ejection fraction of 60-65% (Figure 1). Diastolic parameters indicated impaired relaxation consistent with grade I diastolic dysfunction. No regional wall motion abnormalities were noted, and there was no evidence of intracardiac thrombus, structural valvular disease, or pericardial effusion (Figure 2).

**Figure 3:**
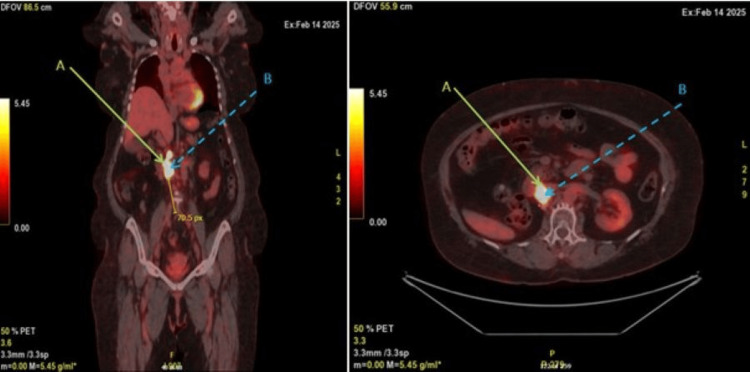
PET scan of the recurrent tumor mass (A) and the IVC mass (B). Left panel: coronal PET image showing a distance of 70.5 pixels from the inferior margin of the IVC mass to the iliac vein bifurcation. Given a pixel spacing of 3.3 mm/pixel, this corresponds to a physical distance of approximately 232.65 mm. Right panel: axial PET image showing the recurrent tumor mass (A) and the IVC mass (B) as a conglomerate. IVC: inferior vena cava

A PET-CT scan identified a hypermetabolic mass at the site of the previous right nephrectomy, extending from 2 cm above the iliac bifurcation to just above the left renal vein. PET findings suggested possible invasion or compression of adjacent structures, including the inferior vena cava (IVC) (Figure 3). The patient was classified as American Society of Anesthesiologists (ASA) physical status IV. Following multidisciplinary evaluation, surgical exploration of the recurrent mass and associated inferior vena cava (IVC) involvement was planned. The operative strategy included IVC exposure, with intraoperative decision-making to determine whether the lesion represented tumor infiltration or thrombus, and whether partial resection or thrombectomy would be required. Coordinated input from urology, vascular surgery, anesthesia, and intensive care teams was essential to ensure optimal intraoperative safety and postoperative recovery.

Anesthetic management

The patient was positioned supine, and comprehensive intraoperative monitoring was established. This included continuous electrocardiography (ECG), non-invasive blood pressure (NIBP), pulse oximetry (SpO₂), invasive arterial pressure monitoring via a radial arterial line, central venous pressure (CVP) monitoring through a catheter placed in the right internal jugular vein, and hourly urine output measurement. In addition, depth of anesthesia was monitored using bispectral index (BIS), while neuromuscular function was continuously monitored using train-of-four (TOF) stimulation via the ulnar nerve, integrated into the Maquet anesthesia machine, to guide neuromuscular blocker dosing.

**Figure 4:**
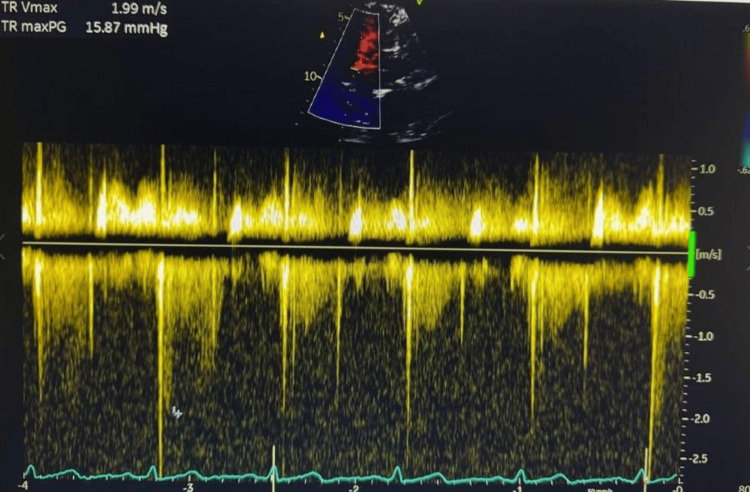
Preoperative continuous-wave Doppler showing tricuspid regurgitation with a peak velocity of 1.99 m/s and pressure gradient of 15.87 mmHg, indicating normal pulmonary artery pressure.

Although preoperative transthoracic echocardiography (TTE) does not offer real-time intraoperative monitoring, it provides valuable baseline information on cardiac function. In this case, preserved right ventricular function and the absence of pulmonary hypertension on TTE supported the use of cautious fluid management during IVC manipulation, reducing the risk of right heart decompensation (Figure 4). Additionally, TTE aided in evaluating the cranial extent of tumor thrombus, contributing to comprehensive perioperative planning in advanced renal cell carcinoma. Controlled ventilation was administered using pressure-regulated volume control (PRVC) mode. Ventilation was adjusted to maintain normocapnia, with end-tidal CO_2_ levels consistently between 35 and 40 mmHg, and oxygen saturation levels were maintained above 95% throughout the procedure.

To reduce intraoperative risks, such as massive bleeding, the blood products were prepared in anticipation of potential activation of the massive transfusion protocol (MTP). Normothermia was maintained using fluid warmers, convective temperature management, forced-air warming, and a low-flow anesthesia circuit with heat-moisture exchange filters to minimize respiratory heat loss. Nitrous oxide was avoided due to the risk of air embolism. Continuous end-tidal carbon dioxide (EtCO₂) and blood pressure monitoring were used as part of our intraoperative strategy to detect any sudden drops suggestive of pulmonary embolism. The surgery lasted approximately 8 hours. Due to the extended operative time and the heightened risk of thrombosis associated with inferior vena cava (IVC) obstruction, mechanical thromboprophylaxis using pneumatic compression devices was employed throughout the procedure.

General anesthesia was induced with intravenous propofol at a dose of 1.2 mg/kg (100 mg), ketamine 0.24 mg/kg (20 mg), fentanyl 100 µg (1.2 µg/kg) remifentanil 1.2 µg/kg (100 µg), midazolam 0.012 mg/kg (1 mg), and rocuronium 0.6 mg/kg (50 mg) to facilitate intubation. Prior to the surgical incision, a bilateral rectus sheath block was administered under ultrasound guidance. A total of 40 mL of 0.375% ropivacaine (20 mL per side) was injected into the fascial plane along the lateral border of the posterior rectus sheath.

Maintenance of anesthesia was achieved using desflurane in an oxygen/air mixture, with an end-tidal concentration corresponding to an age-adjusted minimum alveolar concentration (MAC) of approximately 0.5-0.9. This was part of a multimodal anesthetic approach that included continuous infusions of remifentanil and dexmedetomidine, along with low-dose propofol and a bilateral rectus sheath block. Anesthetic depth was monitored using bispectral index (BIS), which was maintained between 35 and 45 throughout the procedure.

In addition to inhalational anesthesia, a continuous propofol infusion was administered at 1.3 mg/kg/h (total dose: 905 mg), alongside remifentanil at a rate of 0.05 µg/kg/min-0.35 µg/kg/min (total dose: 9.688 mg). Dexmedetomidine was infused at 0.241 µg/kg/h (total dose: 151.36 µg) starting after induction and continued intraoperatively to provide analgesic and opioid-sparing effects. Intravenous paracetamol 1 g was given near the end of the procedure to supplement postoperative pain management. A single 10 mg dose of intravenous oxycodone was administered upon completion of surgery, prior to the patient’s transfer to the intensive care unit (ICU). Neuromuscular blockade was achieved with intermittent bolus doses of rocuronium, totalling 80 mg intraoperatively. Despite the relatively low cumulative dose, neuromuscular function was effectively suppressed, with the train-of-four (TOF) count consistently maintained between 0 and 1 throughout the procedure.

Despite complete inferior vena cava (IVC) obstruction, the patient experienced significant intraoperative blood pressure fluctuations due to IVC manipulations, clamping, and declamping. Hemodynamic support was provided using infusions of phenylephrine (total dose: 5715 µg; approximately 0.85 µg/kg/h) and norepinephrine (total dose: 189.7 µg; approximately 0.28 µg/kg/h). Both agents were titrated to maintain a mean arterial pressure (MAP) of ≥65 mmHg, with real-time adjustments guided by intraoperative surgical events and clinical indicators of perfusion, including urine output, lactate levels, and peripheral perfusion. Central venous pressure (CVP) was continuously monitored; however, due to altered venous return dynamics associated with IVC obstruction, trends rather than absolute CVP values were used alongside clinical markers, such as urine output and lactate, to guide fluid and vasopressor therapy. Nitroglycerin was administered in intermittent boluses, with a total cumulative dose of 0.05 mg, to attenuate hypertensive episodes during tumor and IVC manipulation. An intraoperative insulin infusion was titrated to maintain blood glucose levels below 180 mg/dL.

Intraoperative fluid management was guided by trends in central venous pressure (CVP) and intraoperative feedback from surgeons experienced in complex oncovascular procedures, allowing real-time assessment of venous congestion and bleeding risk. Additionally, intermittent evaluation of urine output, serum lactate levels, and central venous oxygen saturation (ScvO_2_) from blood gas analysis provided important indicators of end-organ perfusion. A total of 10 L of crystalloid and 200 mL of 20% albumin were administered to maintain perfusion. One unit (250 mL) of packed red blood cells (PRBCs) was transfused intraoperatively in response to a drop in hemoglobin from 14.9 to 8.0 g/dL, ongoing surgical blood loss, rising lactate, and vasopressor requirements suggesting impaired oxygen delivery. This single-unit transfusion strategy was guided by dynamic clinical assessment and is consistent with restrictive, goal-directed transfusion practices recommended by current guidelines.

At the end of the procedure, total urine output was 1,400 mL, and estimated blood loss was approximately 500 mL, suggesting adequate renal perfusion and hemodynamic stability during the procedure, based on urine output, low estimated blood loss, and maintained perfusion parameters. Mild, transient metabolic acidosis occurred intraoperatively, evidenced by a lowest pH of 7.29, bicarbonate level of 19.4 mmol/L, and a peak lactate of 2.6 mmol/L. These abnormalities were managed with titrated fluids and hemodynamic support using vasopressors and vasodilators. As shown in Table 1, intraoperative fluctuations in acid-base and metabolic parameters stabilized in the postoperative phase, reflecting adequate perfusion and recovery.

**Table 1:** Arterial blood gas (ABG) trends over time in the perioperative period. This table displays serial ABG measurements at defined intraoperative and postoperative time points, with reference ranges included for each parameter. Key variables, including pH, PCO₂, PO₂, HCO₃-(P), and base excess, are presented to reflect changes in acid-base status, ventilation, and oxygenation throughout the perioperative course. HCO₃-(P): plasma bicarbonate; CtHb: total hemoglobin concentration; SO₂: oxygen saturation; FO₂Hb: fraction of oxyhemoglobin; CtBil: total bilirubin concentration; Base (ECF): base excess in the extracellular fluid

Parameters	Reference range	08:30:00	10:26:00	10:42:00	11:08:00	12:13:00	12:55:00	13:51:00	14:16:00	15:42:00	16:13:00	19:31:00	04:31:00	11:17:00	12:15:00
pH	7.35-7.45	7.331	7.241	7.316	7.282	7.286	7.257	7.222	7.275	7.268	7.291	7.387	7.389	7.381	7.396
PCO_2_	35-45 mmHg	41.7	39.2	43.2	43.5	45.6	46.8	47.3	47.3	47.4	45	36.8	36.9	40.1	39.2
PO_2_	83-108 mmHg	59.6	92.8	104	95.4	94.4	114	108	100	88.7	80	81.2	78.2	64.5	77.6
HCO_3_-(P)	21-28 mmol/L	22	16.8	22	20.5	21.7	20.8	19.4	23.3	21.6	21.7	22.1	22.3	23.8	24
CtHb	11.0-15.0 g/dL	14.9	9.6	10.4	9.9	8	9.2	9.4	9.2	9	9.2	9.6	9.2	8.7	11.9
SO_2_	95-99%	94.4	96.9	98.6	98	98.1	98.7	98.4	98.3	97.3	96.4	97.5	96.9	93.7	96.4
FO_2_Hb	95-98%	93.9	95.3	97	96.3	96.2	96.8	96.4	96.1	95.2	94.4	95.6	95.3	92.1	95.3
CtBil	0-1.2 mg/dL	0	0	0	0.4	0.4	0.2	0.6	0.4	0.4	0.3	0.6	0.3	0.3	0.4
K	3.4-5.0 mmol/L	4	3.7	4.3	4.3	4.4	4.4	4	3.9	3.6	3.7	3.9	3.9	4	3.5
Na	134-143 mmol/L	146	146	147	145	146	146	145	148	148	147	146	145	146	140
Ionized calcium	1.15-1.29 mmol/L	1.26	1.2	1.12	1.13	1.11	1.11	1.09	1.16	1.14	1.19	1.21	1.18	1.21	1.18
Cl	97-108 mmol/L	114	121	116	116	115	116	114	116	117	116	112	114	114	107
Glucose	60-100 mg/dL	110	165	161	203	180	200	229	173	179	150	167	144	133	145
Lactate	0.5-1.6 mmol/L	1	1.1	1.4	1.6	2.4	2.1	2.5	2.9	2.6	1.6	2.4	1.5	1.3	0.9
Base (ECF)	mmol/L	3.9	10.5	4.1	6.2	4.9	6.3	8.3	3.6	5.3	4.9	2.9	2.7	1.3	0.9
Time points		Pre-induction	Intraoperative-1	Intraoperative-2	Intraoperative-3	Intraoperative-4	Intraoperative-5	Intraoperative-6	Intraoperative-7	Intraoperative-8	Intraoperative-9	ICU-1	ICU-2	ICU-3	1CU-4

Surgical details

**Figure 5:**
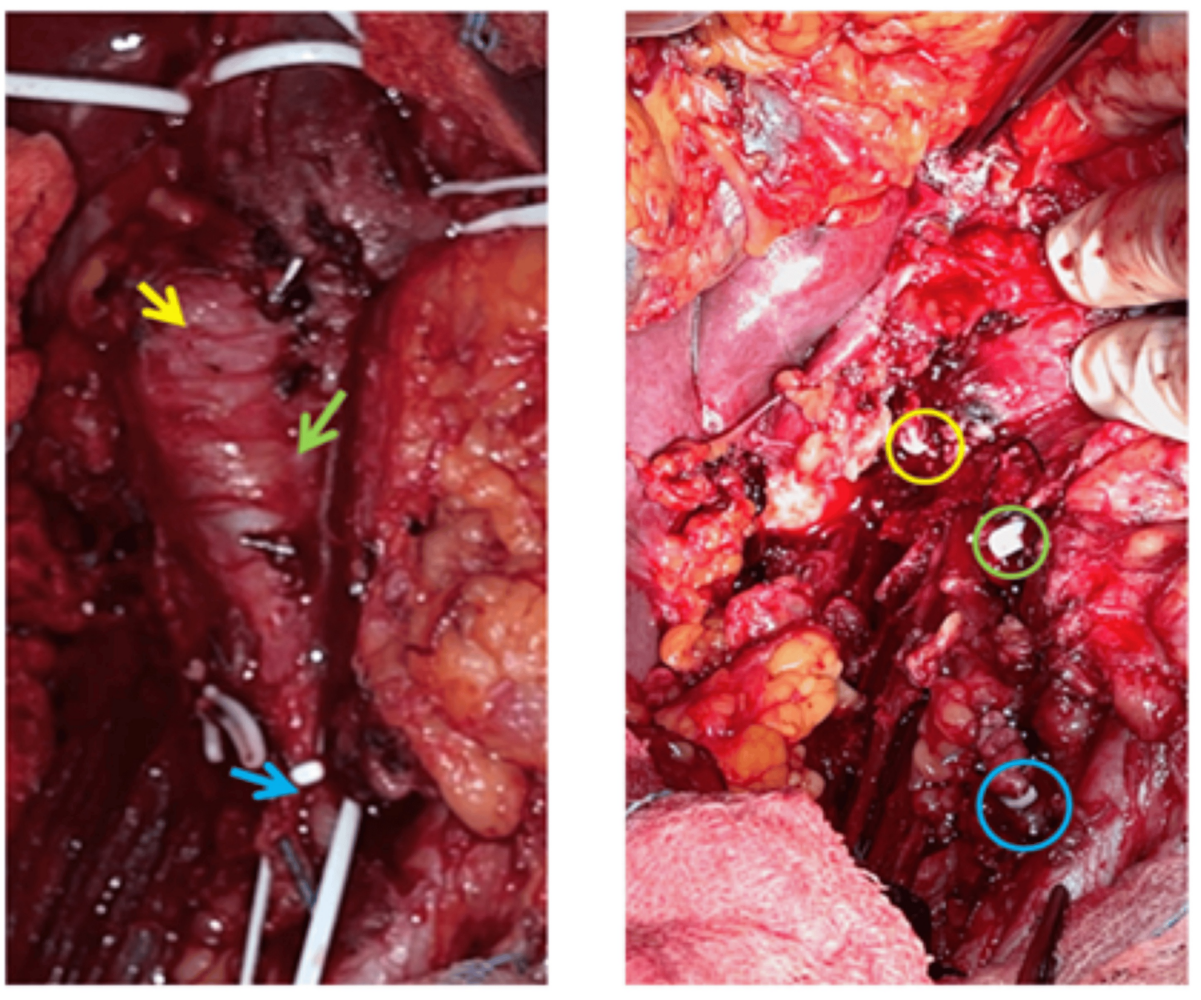
Intraoperative images during oncological surgery before and after resection of the tumor mass and inferior vena cava. Left panel: intraoperative image showing recurrent tumor at the nephrectomy site (yellow arrow) and IVC tumor thrombus extending cranially to 2.3 cm above the iliac bifurcation (green arrow). The distal IVC (blue arrow) appears tumor-free. Right panel: post-resection view with tumor-free renal bed (yellow circle), cranial (green circle), and caudal (blue circle) IVC ligation sites. The IVC was resected without reconstruction. IVC: inferior vena cava

Following midline laparotomy, the vascular team joined the urology team for coordinated resection. After mobilizing the bowel and retroperitoneum, the inferior vena cava (IVC) was exposed and controlled distal to the thrombus. The left renal vein was also exposed and assessed for tumor involvement. The IVC was clamped approximately 1 cm above the orifice of the left renal vein, where the tumor extension terminated. Intraoperative findings confirmed complete tumor invasion of the IVC from 2 cm above the iliac bifurcation up to the left renal vein orifice. En bloc resection of the involved IVC segment, tumor thrombus, and surrounding tissue was performed. The left renal vein was found to be uninvolved, and the urology team, noting preserved left renal function, recommended no further intervention. No reconstruction was undertaken; the vein was ligated using vascular clips. The proximal and distal IVC stumps were closed (Figure 5). Hemostasis was secured, and the surgical field was irrigated. Drains were placed in the retroperitoneum and subcutaneous layers before layered closure. The specimen was sent for histopathological analysis.

Following surgery, she was transferred intubated to the intensive care unit (ICU), where sedation was maintained with low-dose infusions of remifentanil and propofol. Postoperative monitoring revealed stable hemodynamics, adequate urine output, and no significant abnormalities on imaging. Sedation was gradually weaned, and the patient was successfully extubated 5 hours postoperatively in the ICU without complications.

## Discussion

Although the patient had a chronically obstructed inferior vena cava (IVC), significant intraoperative blood pressure swings were observed. This phenomenon can be attributed to multiple factors. First, while collateral venous circulation (e.g., via the azygos and lumbar veins) develops over time, these channels are low-pressure, thin-walled vessels that are easily compressed or disrupted by surgical retraction or tumor manipulation [6,7]. In this case, fluctuating venous return and subsequent hemodynamic instability were observed during recurrent tumor mobilization and IVC manipulation, likely due to transient disruption of collateral flow. Second, dissection near the IVC may stimulate the autonomic nervous system, leading to sympathetic surges or vagal responses that contribute to hemodynamic instability, particularly in the presence of tumor adherence to vascular structures [8]. Additionally, dynamic tumor compression or release may intermittently impact residual flow or collateral routes, leading to sudden preload changes [9]. Finally, although the IVC was completely obstructed, clamping and unclamping at levels above the obstruction, such as the renal veins, can still influence venous drainage from organs like the kidney and liver, releasing vasoactive substances and causing abrupt hemodynamic shifts [10,11]. These observations underscore the need for vigilant monitoring and flexible anesthetic strategies even in cases of total IVC occlusion.

The intraoperative drug dosing, when adjusted for body weight, remained within or below standard anesthetic reference ranges: propofol at 1.3 mg/kg/h (reference range: 4-12 mg/kg/h), remifentanil at 0.24 µg/kg/min (reference range: 0.1-0.3 µg/kg/min), and rocuronium at 0.3 mg/kg/h (reference range: 0.6-0.72 mg/kg/h) [12]. The reduced anesthetic requirements were attributed to the synergistic effect of a multimodal anesthesia approach incorporating a bilateral rectus sheath block. This regional technique likely enhanced somatic nociceptive blockade, enabling minimization of systemic anesthetic and opioid use while maintaining hemodynamic stability [5].

The anesthetic regimen combined inhalational desflurane with intravenous propofol, dexmedetomidine, and opioid infusions, supplemented by regional blockade, in accordance with principles outlined in Enhanced Recovery After Surgery (ERAS) protocols [13]. Although this was not a total intravenous anesthesia (TIVA) technique, the integration of balanced anesthesia with a bilateral rectus sheath block aligns with current evidence supporting regional techniques. Such approaches have been shown to improve postoperative pain control, reduce systemic opioid requirements, and facilitate early extubation and intensive care unit (ICU) discharge [14]. Our experience reinforces the value of opioid-sparing, multimodal strategies even when short-acting intraoperative opioids, such as remifentanil, are employed in major abdominal and vascular procedures, particularly in high-risk surgical patients. The limited postoperative requirement for opioids, including a single 10 mg dose of intravenous oxycodone, further supports the effectiveness of this approach in enhancing recovery while maintaining adequate analgesia [15].

The patient’s uneventful postoperative ICU course supports the success of the perioperative strategy. She was extubated without complications. Only low-dose norepinephrine was required for the first 4 hours postoperatively, and a single 10 mg dose of furosemide was administered in response to transient low urine output, with good effect. No further interventions were necessary. Although patient-controlled analgesia (PCA) with morphine was prescribed postoperatively, the patient did not utilize it and required no additional analgesia during the first 24 hours after extubation. She was discharged from the ICU after 15 hours, pain-free and hemodynamically stable.

Recent literature reinforces the clinical relevance of our findings. Zhang et al. and Li et al. noted that patients undergoing IVC thrombectomy frequently experience significant hemodynamic fluctuations due to manipulation of venous collaterals and tumor burden, particularly in the presence of chronic obstruction, necessitating vigilant intraoperative monitoring and readiness for vasoactive support [3-8]. Kaur et al. reported that dynamic preload changes, venous stasis, and intra-abdominal manipulation contribute to unpredictable blood pressure variations in such cases, underscoring the importance of invasive monitoring and timely pharmacologic intervention [10].

Our approach, which incorporated balanced anesthesia with agents, such as propofol, remifentanil, desflurane, and adjunctive dexmedetomidine, reflects the growing consensus on the benefits of opioid-sparing strategies. Dexmedetomidine has been shown to provide stable sedation, reduce sympathetic responses, and facilitate smoother emergence from anesthesia, especially when used alongside short-acting opioids like remifentanil [5].

The bilateral rectus sheath block was performed using 0.375% ropivacaine (20 mL per side), which typically provides analgesia for 6-12 hours. While isolated reports suggest that effects may occasionally extend up to 18-20 hours. Several studies and meta-analyses have shown that systemic (IV) dexmedetomidine prolongs the analgesic effects of fascial plane blocks, including rectus sheath block (RSB), by enhancing central modulation of pain. Its use may have contributed to the prolonged postoperative comfort observed in our patient despite the expected duration of single-shot ropivacaine [14]. Kianian et al. also highlighted that multimodal analgesia improves hemodynamic stability, reduces ICU stays, and enhances early recovery [5]. Recent ERAS-focused studies confirm that early extubation in high-risk vascular surgery patients is not only feasible but also associated with better pulmonary outcomes and reduced ICU time [16]. In contrast, a retrospective cohort study by Hua et al. investigated the incidence and risk factors of myocardial injury and acute kidney injury (AKI) in patients undergoing radical nephrectomy with IVC thrombectomy. The study found that myocardial injury occurred in 37.8% and AKI in 42.7% of patients postoperatively. These complications were associated with worse perioperative outcomes, including longer hospital stays and increased morbidity [17]. These findings underscore the importance of optimizing perioperative management to reduce complications and ICU length of stay in RCC patients undergoing complex surgeries involving the IVC. Our patient’s smooth ICU course, minimal opioid needs, and stable hemodynamics illustrate the practical benefit of this evidence-based, multidisciplinary approach.

## Conclusions

This case highlights the effectiveness of a carefully planned balanced anesthetic approach combining inhalational and intravenous agents with regional analgesia in the context of complex oncovascular surgery. Despite the presence of complete inferior vena cava (IVC) obstruction and multiple comorbidities, intraoperative hemodynamics remained well controlled, and anesthetic drug requirements were kept at the lower end of standard dosing ranges. The use of bilateral rectus sheath block as part of a multimodal, opioid-sparing strategy provided effective somatic analgesia, minimising the need for high-dose opioids and deep neuromuscular blockade. This facilitated early extubation, rapid postoperative recovery, and a complication-free intensive care unit (ICU) stay. These findings are consistent with Enhanced Recovery After Surgery (ERAS) principles and support the role of regional techniques and individualized anesthetic management in improving outcomes in high-risk abdominal vascular procedures.
